# Osteoimmunology of Fracture Healing

**DOI:** 10.1007/s11914-024-00869-z

**Published:** 2024-04-15

**Authors:** Kristin Happ Molitoris, Mingjian Huang, Gurpreet Singh Baht

**Affiliations:** Department of Orthopaedic Surgery, Duke Molecular Physiology Institute, Duke University, 300 North Duke Street, Durham, NC 27701 USA

**Keywords:** Bone fracture, Fracture callus, Immune cells, Inflammation, Hematopoietic cells

## Abstract

**Purpose of Review:**

The purpose of this review is to summarize what is known in the literature about the role inflammation plays during bone fracture healing. Bone fracture healing progresses through four distinct yet overlapping phases: formation of the hematoma, development of the cartilaginous callus, development of the bony callus, and finally remodeling of the fracture callus. Throughout this process, inflammation plays a critical role in robust bone fracture healing.

**Recent Findings:**

At the onset of injury, vessel and matrix disruption lead to the generation of an inflammatory response: inflammatory cells are recruited to the injury site where they differentiate, activate, and/or polarize to secrete cytokines for the purposes of cell signaling and cell recruitment. This process is altered by age and by sex.

**Summary:**

Bone fracture healing is heavily influenced by the presence of inflammatory cells and cytokines within the healing tissue.

**Graphical Abstract:**

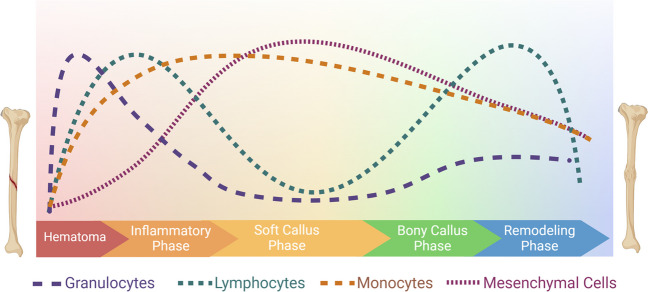

## Introduction

Inflammation plays a critical role in robust bone fracture healing. Dysregulated inflammation, as seen in cases of advanced age, diabetes, obesity, and autoimmune disease, is often associated with non-union, delayed healing, and other complications [1–3]. In this review, we will discuss the different immune cells and their role in the stages of fracture healing. We also discuss how these processes change with advanced age and with sex.

## Progression of Fracture Healing

**Fig. 1:**
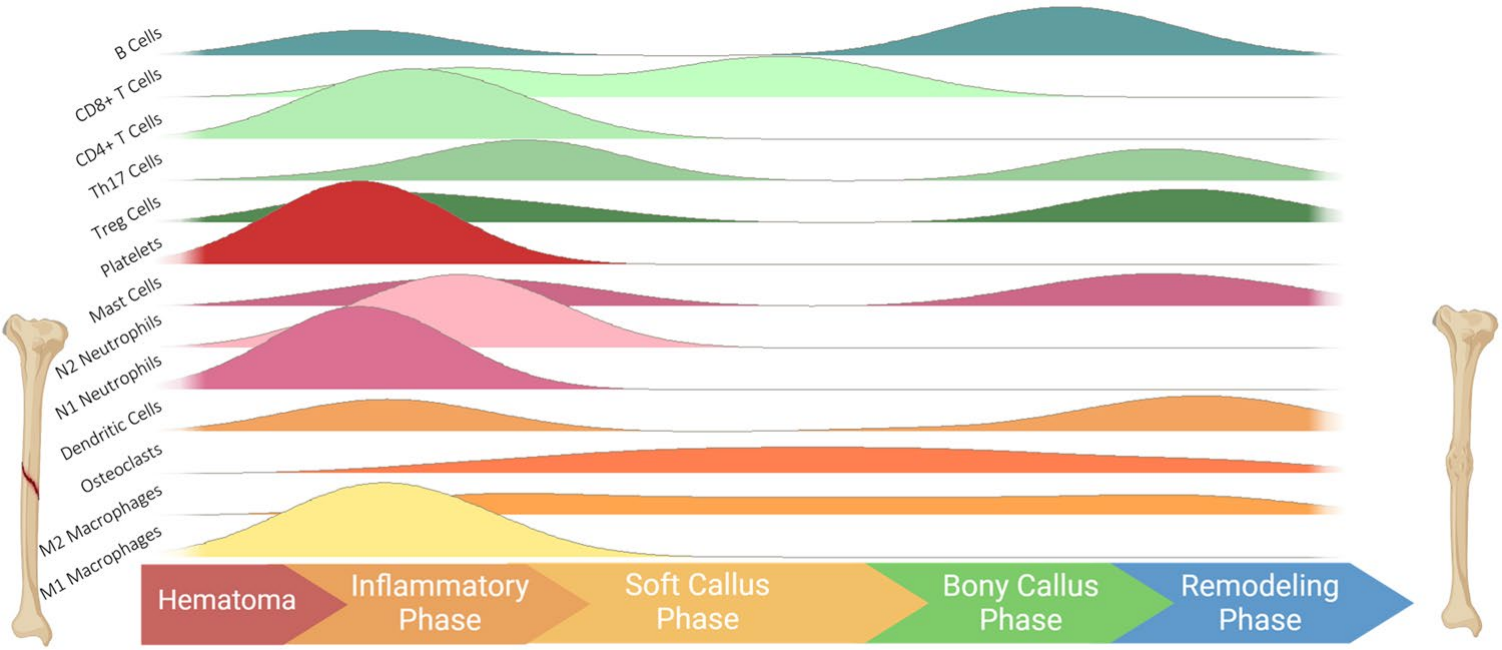
Timeline of immune cell activity during the course of bone fracture healing

Fracture healing is a multiphasic process (Fig. 1). Bone vasculature is disrupted upon injury and immune cells from peripheral blood are recruited to the site. A hematoma is formed during this first inflammatory stage and immune cells from bone marrow are now recruited and activated. The hematoma is then reorganized and a fibrin-rich scaffold of granulation tissue forms. This scaffold is turned over into a cartilaginous callus as capillaries invade and recruited tissue-resident mesenchymal progenitors differentiate to chondrocytes. The end of this stage is marked by terminal differentiation of chondrocytes (chondrocyte hypertrophy) and calcification of the cartilaginous callus. Osteochondral progenitor cells and tissue-resident osteoblasts (OBs) then enter the callus to deposit new woven bone, replacing the cartilaginous callus with a bony callus. Finally, remodeling of the callus occurs and laminar bone replaces the woven bone [4, 5].

It has become well established within the last decade that the immune system plays a critical role in the process of bone fracture healing—this field is often termed *osteoimmunology* [6]. Innate immune cells derived from the bone marrow are present in the callus minutes after the injury occurs. Their secreted inflammatory cytokines initiate signaling cascades that affect mesenchymal cells and that are necessary for cell-to-cell crosstalk. This initial inflammation is critical for healing. Importantly, fracture healing is diminished and often associated with complications including non-union or delayed union when these inflammatory processes are dysregulated, as seen in advanced age [7]. Adaptive immune cells take over during healing after this initial inflammatory cascade. Interestingly, suppressing the adaptive immune system has been correlated with accelerated healing yet patients treated with general immunosuppressants show delayed healing, indicating that the immune system coordinates a carefully timed signaling cascade for optimal resolution [8, 9]. In this review, we will highlight the role that immune cells play in the process of fracture healing.

## Hematopoietic Cells

*Hematopoietic stem cells* (HSCs) originate in the bone marrow and give rise to all cells of the immune system. Co-localized mesenchymal cells (mesenchymal progenitors and differentiated osteoblasts, adipocytes, and chondrocytes) produce an extracellular matrix network of proteins such as collagen, fibronectin, laminin, proteoglycans, and thrombospondin and secrete cytokines such as interleukins (ILs), GM-CSF, Flt3 ligand, Ang-1, and CXCL3 [10]. Together, these components from mesenchymal cells modulate HSC actions such as self-renewal, viability, expansion, differentiation, and mobilization, thereby impacting all downstream effects [11]. HSCs differentiate into two primary progenitor cell types—common myeloid progenitors (CMPs) and common lymphoid progenitors (CLPs).

### Common Myeloid Progenitors

**Fig. 2:**
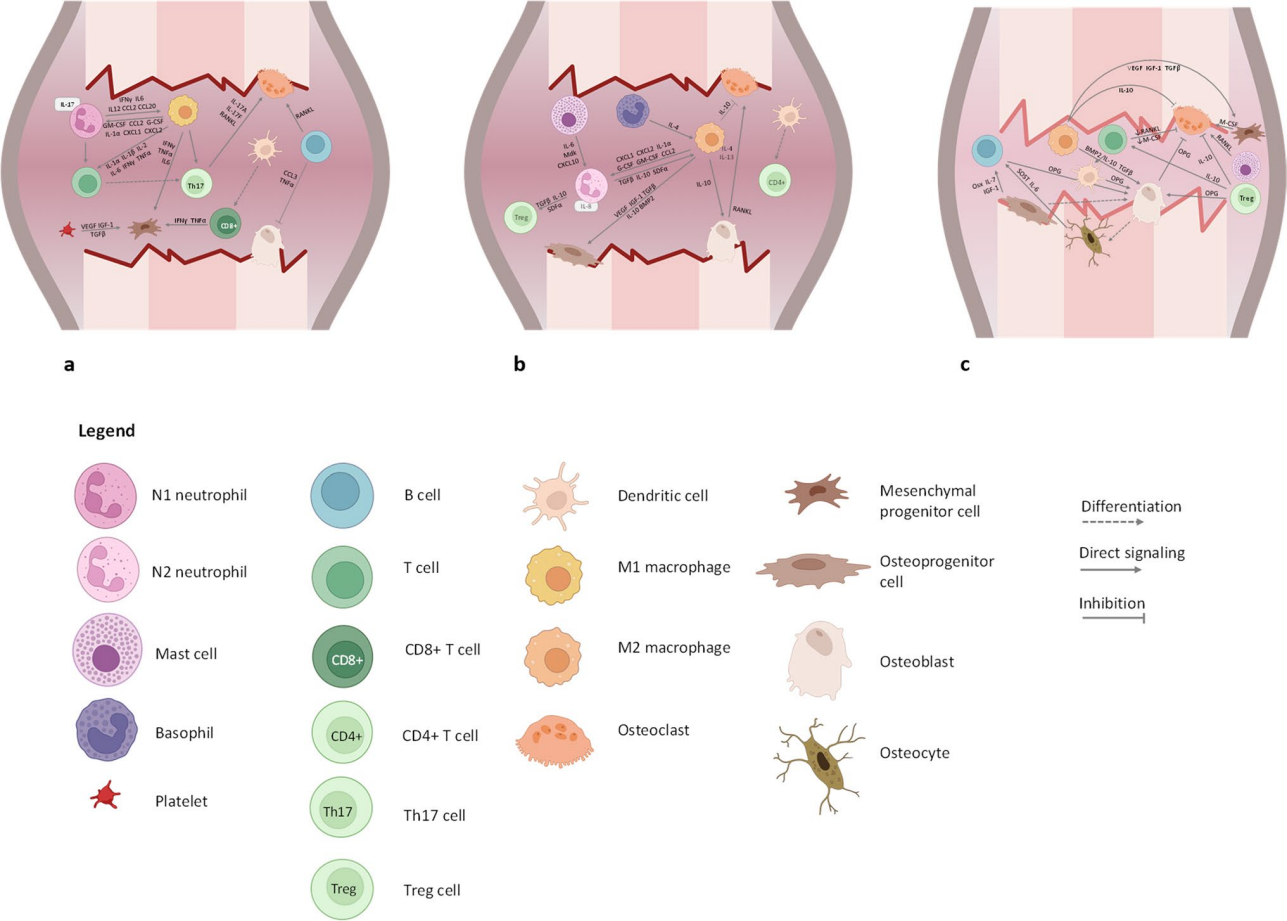
**a** Immune response during hematoma formation at the fracture site. **b** Immune response during the inflammatory phase at the fracture site. **c** Immune response in the soft and bony callus formation and remodeling phases of the fracture

CMPs give rise to monocytic cells such as macrophages, osteoclasts, and dendritic cells, as well as granulocytic cells such as neutrophils, mast cells, megakaryocytes, platelets, basophils, and eosinophils. All of these cells play important roles in bone biology and repair (Fig. 2).

#### Macrophages

Macrophages are terminally differentiated monocytic cells able to polarize through a multitude of pro-inflammatory and anti-inflammatory cell fates. In response to fracture, macrophages are recruited to the injury site and raise an inflammatory response that is critical for robust healing and that is followed by an anti-inflammatory response [12, 13].

Bone-resident macrophages/osteal macrophages (often termed “Osteomacs”) appear during neonatal development and are maintained throughout the mammalian lifespan. They line the endosteal bone surface and play an important anti-inflammatory role in bone homeostasis [14, 15••]. Osteomacs have been found to interact with megakaryocytes and OBs to maintain HSC function but respond in a limited manner to exogenous stimuli and play a relatively minor role in fracture healing relative to their bone marrow–derived counterparts [16, 17].

Bone marrow–derived macrophages (BM MΦ) are among the first cells to be recruited to the fracture site. At this early time period, the pro-inflammatory environment causes polarization toward a pro-inflammatory “M1” phenotype. M1 macrophages do not rely on a steady oxygen supply but rather thrive in a hypoxic environment, making them ideal first-responders in the disrupted vasculature of the hematoma. They secrete CXCL1, CXCL2, IL-1α, and CCL2 for neutrophil recruitment and G-CSF and GM-CSF for neutrophil maintenance [18]. They also secrete pro-inflammatory cytokines such as IL-1α, IL-1β, IL-2, IL-6, IFNγ, and TNFα, which activate T cells and recruit mesenchymal progenitor cells. In turn the IL-1 family of cytokines drive endothelial cells to secrete VEGF and stimulate angiogenesis within the fracture callus [19, 20].

Following this initial pro-inflammatory stage in healing, the number of M1 macrophages subsides and a switch in favor of anti-inflammatory “M2” macrophages is observed. The M2 macrophage population grows through recruitment of monocytic BM MΦs and Osteomacs and through polarization of M1 macrophages to M2 macrophages. This switch between the M1/M2 subtype is crucial for successful fracture healing [21]. Prior to this switch, M1 macrophages induce RANKL expression by osteoblasts and T cells causing osteoclastogenesis that is necessary for remodeling of the healing fracture callus. Conversely, M2 macrophages secrete IL-10 to inhibit osteoclast function during later stages of healing [21, 22]. During remodeling, M2 macrophages secrete TGFβ, VEGF, and IGF-1 to promote revascularization and BMP-2, IL-10, and TGFβ to induce osteoblast differentiation [23].

BM MΦs play a critical role in bone repair: ablation of macrophages is associated with decreased bone deposition within the fracture callus and higher rates of non-union [13, 14, 24].

#### Osteoclasts

Osteoclasts (OCs) are monocytic cells that resorb the bone matrix and play a critical role in bone remodeling. Mononucleated OC precursors reside on bone surfaces and fuse to form multi-nucleated OCs upon activation. The inflammatory environment of the initial stage of bone fracture healing activates osteoclasts to clear out damaged tissue in concert with macrophages. Activated osteoclasts are later tasked to resorb the cartilaginous callus and subsequently the bone callus [25].

During these callus-remodeling stages, RANKL serves as an obligate signaling molecule needed for OC activation and proliferation [26, 27]. RANKL is secreted by a number of cell types within the fracture callus but osteoblasts serve as the major source. RANKL binds the OC cell-surface receptor RANK and this RANKL-RANK binding is critical for both osteoclast formation and recycling [25, 26]. Importantly, RANKL alone is not sufficient for OC activation as M-CSF is also required. M-CSF is produced by a variety of mesenchymal cell types including endothelial cells, myoblasts, epithelial cells, fibroblasts, and OBs. More specifically, Inoue et al. identified adipoq-lineage cells are likely the major source of M-CSF within the bone marrow [28].

OBs secrete a decoy receptor to RANKL to balance osteoclast activation: osteoprotegerin (OPG) is able to bind and sequester RANKL, preventing osteoclast activation. While OC-OB crosstalk is perhaps the most studied, OCs have been shown to act as antigen-presenting cells to activate and control T cell response [29–31]. However, more work must be done to fully elucidate the immunogenic function of osteoclasts during fracture healing.

#### Dendritic Cells

The role of dendritic cells (DCs) in fracture healing is still being unraveled; however, they seem to primarily function as signaling cells. DCs are present in homeostatic bone; their population size peaks in the inflammatory phase of fracture healing then significantly decreases, returning to baseline by the bone callus/remodeling phase [1, 20]. DCs can function as both innate and adaptive immune responders [32]. Serving as innate immune cells, DCs initiate CD8 + T cell responses for the initial inflammation upon fracture injury [33]. Serving as adaptive immune cells, DCs produce OPG to diminish osteoclast resorptive activity during later stages of healing [34].

Through secretion of cytokines, DCs can reactivate primed CD4 + T cells to determine T cell fate (Treg/Th1/Th2/Th17/Tfh cells) [35]. Interestingly, using single-cell RNA sequencing (scRNA-seq), Avin et al. found that CD14 + DCs were a macrophage-like cell population, present exclusively in human patients with non-union and not in healthy controls [36]. However, more work remains to be done to delineate the role of DCs in bone fracture healing.

#### Granulocytes

Granulocytes are a group of cells that differentiate from CMPs yet differ in morphology and function from monocytes. These cells secrete growth factors and cytokines in response to bone injury leading to recruitment of immune and mesenchymal progenitor cells and revascularization [4, 37]. Granulocytes give rise to neutrophils, mast cells, megakaryocytes, platelets, basophils, and eosinophils. The bulk of the studies of granulocytes and their role in bone fracture healing have focused on neutrophils which are among the first and most abundant immune cells to arrive at the fracture site, while megakaryocytes, platelets, basophils, eosinophils, and mast cells are much less populous during fracture healing [38].

*Neutrophils* polarize into different subtypes during inflammatory events such as fracture healing. Similar to M1 macrophages, neutrophils have an anaerobic glycolytic pathway to function in the oxygen-deprived microenvironment of the hematoma. Pro-inflammatory N1 neutrophils are either recruited by IL-17 directly or converted from TNF-α-recruited undifferentiated neutrophils, respond immediately to M1 macrophages, and secrete IFNγ, IL-6, IL-12, CCL2, and CCL20. These cytokines promote the differentiation of CD4 + T cells into Th1 and Th17 cells (discussed more below). Anti-inflammatory N2 neutrophils are polarized by IL-8 and switch their secretomes to TGF-β, IL-10, and SDF-1α which in turn promotes Tregs and alternatively activated M2 macrophages [19, 39–41].

Much less is known about the function of less abundant granulocyte-derived cells within the fracture callus. However, studies thus far have indicated these cells may play important roles during bone homeostasis and repair.

*Mast cells* are present in small numbers at the fracture site and act immediately after injury to recruit immune cells such as neutrophils to the site via the release of inflammatory cytokines such as IL-6, Mdk, and CXCL10. In later stages of healing, mast cells increase in number and promote OC formation [42–44].

*Megakaryocytes* (MKs) have not been directly studied for their role in fracture healing. There exists, however, a small body of literature that examines the role of MKs in normal bone homeostasis. MKs act via cell-to-cell contact and secrete factors to inhibit osteoclastogenesis, induce OB expression of OPG and collagen, and decrease OB expression of RANKL [45, 46]. Meijome et al. showed that, while OPG knockout mice develop decreased trabecular thickness and trabecular bone volume, an increase in MK population reversed this adverse phenotype [47]. While studies such as this present a role for MKs in bone biology, more work needs to be done to elucidate the role of MKs in fracture healing.

*Platelets*, a non-nucleated cell product of MKs, have been found to be beneficial for fracture healing. Platelet-rich plasma treatment in multiple types of human fractures was found to overall decrease time of healing and increase bone mineral density (reviewed in Xu et al. [48]). Platelets are critical for the fibroblastic cell proliferation in the initial inflammatory phase of fracture healing. Platelets secrete PDGF, VEGF, TGFβ, and IGF-1 to promote mesenchymal progenitor cell proliferation and differentiation [3, 37]. Further studies are required to better understand the mechanistic role of platelets in fracture healing and to identify potential benefits of platelet-rich plasma in bone regeneration.

*Basophils* are the least abundant type of granulocyte and primarily respond to allergens. However, they have also been shown to contribute to tissue fibrosis and regeneration. Basophils can activate fibroblasts in vitro and are an important source of IL-4 in inflammation, signaling to activate M2 macrophages [49]. Their role in fracture healing has yet to be investigated.

*Eosinophils* are a rare population of granulocytes. Eosinophils have been shown to produce the heme-containing enzyme eosinophil peroxidase which is able to inhibit osteoclast differentiation from monocyte precursors [50]. However, more work needs to be done to elucidate the role of these granulocytes in fracture healing. As techniques such as single-cell RNA sequencing become more widely available and less expensive, it may finally be feasible to look at populations that are otherwise challenging to study.

### Common Lymphoid Progenitors

Monocytes and granulocytes are innate responders and serve to support and signal to the adaptive cells; the strongest adaptive immune response in the body is coordinated by lymphocytes. In recent years, much focus in osteoimmunology has been placed on the relationship between lymphocytes and the crosstalk that occurs between lymphocytes, myeloid cells, and mesenchymal cells. Lymphocyte numbers within the region initially decrease after bone injury but rebound near the end of the inflammatory phase ﻿[51•, 52, 53]. CLPs terminally differentiate into T cells and B cells, each with their own subset groups. Both T cell and B cells are increasingly being recognized as crucial for both normal bone health and fracture healing (Fig. 2).

#### T Cells

T cells are characterized by their T cell receptors as either conventional αβ- or unconventional γδ T cells. Conventional T cells further differentiate into CD8 + (“cytotoxic”), CD4 + (“helper”), regulatory (Tregs), or follicular helper (Tfh) T cells.

Cytotoxic CD8 + T cells are the initial, pro-inflammatory T cell population present in the fracture hematoma [19]. This population produces TNFα and IFNγ to recruit the next wave of cells necessary for fracture healing and then rapidly decrease in population to allow for the next stage of repair.

Helper CD4 + T cells are characterized by their cytokine profiles and further differentiate into Th1, Th2, Th9, Th17, or Th22 cells depending on stimuli. Th1 and Th2 can produce cytokines that can act as both pro- and anti-inflammatory factors depending on the timing of secretion during fracture healing; they secrete IFNγ, IL-4, IL-13, IL-18, and IL-33 among others which promotes bone formation by stimulating osteoblast differentiation and by inhibiting osteoclast activity [54, 55]. Th9 cells express IL-9 and while the role of IL-9 in fracture healing is yet unknown, we have recently reported IL-9 to be present within the fracture callus and Elyaman et al. have reported that IL-9 initiates CD4 + cell differentiation to Th17 cells [51•, 56, 57]. Within the fracture, Th17 cells are pro-inflammatory, expressing high levels of IL-17A, IL-17F, and RANKL, and promote the differentiation of osteoclasts to accelerate bone reabsorption [58]. Th22 cells have not been studied in the context of fracture but are known to produce IL-22 and to promote osteoclast differentiation in other bone disorders [59].

Tregs increase in population during development of the soft callus and inhibit osteoclastogenesis [60]. There are two populations of Tregs within the fracture callus—natural Tregs and inducible Tregs. Natural Tregs maintain normal bone homeostasis and inhibit excess osteoclastogenesis via cell-to-cell contact. In fracture healing, inducible Tregs regulate osteoclastogenesis through secretion of inhibitory cytokines such as IL-10; this causes other T cells to suppress secretion of RANKL and M-CSF while upregulating OPG expression [61, 62].

Tfh cells act primarily in the lymph node germinal center closest to the fracture to drive B cell maturation and trafficking [63, 64]. The role of Tfh cells within the fracture callus or during fracture healing has not yet been reported in the literature.

IL-17 is secreted by T cells and recognized to be a critical signaling molecule in fracture healing. Th17 cells from the gut are immediately recruited to the fracture site by the release of sphinogesin-1-phosphate to produce IL-17. Concurrently, IL-23 from DCs activates localized γδ-T cells in the fracture to produce IL-17A. Both populations of IL-17-producing T cells are necessary for proliferation and differentiation of OBs [65–67]. The balance between Th17 and Treg cells is a major modulator of bone formation [58, 68]. While this ratio was first noticed in the context of bone diseases, Dai et al. identified that during the initial stages of fracture healing the Treg population decreases drastically while the Th17 population increases, correlating with a spike in IL-17 [69]. Both T cell subsets arise from immature CD4 + T cells; however, the inflammatory Th17 subtype is driven by signals from M1 macrophage which are among the first cells to arrive at the injury site [70]. Recent work has identified that altering the Th17/Treg ratio balance impacts healing—decreasing Th17 cells and increasing Tregs improved bone repair by inhibiting pro-inflammatory effects of T cells such as IL-6 production [71].

#### B Cells

Naïve B cells are derived from CLPs and further differentiate into either memory B cells or plasma cells. B cells are recruited to the fracture site at the onset of injury, are rapidly depleted by the end of the inflammatory phase, and then sharply increase in number during the hard callus phase to levels well above baseline [51•, 53]. B cells drive early osteoclastogenesis by producing RANKL and inhibit OB differentiation through CCL3 and TNFα expression [72]. B cells are prolific cytokine producers in the fracture callus; they express IL-10 to limit proinflammatory signaling and to reduce the early inflammatory response [3]. Interestingly, Yang and colleagues found that in delayed healing patients, IL-10 from B cells was downregulated compared to normal healing patients during early inflammation [73]. As discussed about T cells above, B cells also have a regulatory subtype (Bregs) that is able to stimulate Tregs and to suppress CD4 + expression of TNFα, IFNγ, and IL-2 and CD8 + expression of TNFα and IFNγ from CD8 + T Cells. Breg dysfunction has been associated with delayed union or non-union patients [73, 74].

Signaling to B cells also plays a role in fracture healing—IGF-1, OSX, IL-7, and CXCL12 produced by osteoprogenitor cells are critical for bone marrow B cell lymphopoiesis [75]. Furthermore, T cells signal to B cells to induce OPG expression [76, 77].

#### Plasma Cells

Plasma cells are a subset of B cells. The role of plasma cells in the context of fracture healing has been minimally studied but much work has been done on understanding the functioning of neoplastic plasma cells in bone in the context of multiple myeloma. Multiple myeloma patients are at higher risk for bone fractures due to malignant plasma cells over-activating OCs and suppressing OBs [78]. Likewise, healthy plasma cells in the fracture callus also have the ability to influence the OB/OC ratio as Li et al. found that plasma cells secreted high amounts of OPG in the homeostatic bone [77]. Future work will delineate the role of plasma cells in bone fracture healing.

## Effect of Age on Fracture Healing

Advanced age is associated with increased rates of delayed union or non-union, and fracture calluses have lower bone volume, structural stiffness, and force to refracture [51•, 79, 80]. These morphological changes are due to age-associated changes in the cell populations present at the injury site as well as age-associated alterations in signaling from these cells. To reverse this phenomenon, Xing et al. first reported that replacement of aged bone marrow HSC’s with young HSC’s was sufficient to rejuvenate bone fracture healing and reverse the aged phenotype [81••].

We and others have shown a homeostatic low-grade inflammatory profile in aging, referred to as *inflammaging* [51•, 79, 80, 81••, 82–84]. Aged OBs, OCs, and macrophages have all been shown to express senescent markers such as p16 and β-galactosidase, reducing their ability to proliferate. Recent work using senolytics indicates that removal of senescent cells in aged mice could improve bone health and aged fracture healing [85, 86]; however, more work must be done to better identify the mechanistic underpinnings at hand.

The inflammation that occurs post-fracture is even slower to resolve with age. We have recently reported this protracted inflammation to be accompanied by altered hematopoietic cell population ratios and altered production of cytokines [51•, 87]. Furthermore, aged HSCs have been shown to have lower regenerative potential and skewed differentiation toward the myeloid lineage instead of the lymphoid lineage [88]. It is not surprising that we found lower numbers of CD4 + T cells and Tregs in aged fracture calluses, whereas the number of myeloid (both granulocyte and monocyte) progenitors was increased [51•]. Interestingly, CD8 + T cells did not decrease in quantity in aged mice compared to young mice [7, 51•, 80, 89]. Inflammaging has also been associated with increased macrophage polarization toward M1 pro-inflammatory macrophages rather than M2 anti-inflammatory macrophages. These macrophages have a reduced capacity for proliferation and are less responsive to GM-CSF. In studies using aged mice, lowering the level of M1 macrophages led to improved fracture healing [3, 87].

In our recent work, we also investigated cytokine profiles prior to and during different stages of fracture healing [51•]. We found that of these tested within the fracture callus, cytokines could be categorized into three groups: (1) *age-accumulated cytokines* (IL-1β, IL-9, IFNγ, and CCL3/MIP-1α), (2) *female-specific age-accumulated cytokines* (IL-2, TNFα, TNFR1, Il-4, and IL-10), and (3) *fracture response cytokines* (CXCL1/KC-GRO, CXCL2/MIP-2, IL-6, CCL2/MCP-1) [51•]. In general, this work in addition to that of others indicates pro-inflammatory cytokines such as IL-1β, IL-6, IFNγ, TNFα, and C-reactive protein (CRP) are more highly expressed in aged patients and associated with an increased risk for fracture [3, 51•, 82, 90, 91]. These inflammatory molecules represent potential points of intervention to improve fracture healing outcome.

## Effect of Sex on Fracture Healing

It is well established that males and females have different outcomes in regard to bone fracture healing [92]. Estrogen (17β-estradiol; E2), the primary sex hormone in females, impacts bone maintenance and growth, and estrogen receptors have been found on OBs, OCs, and osteocytes [93]. It is therefore not surprising that changes in bone health are even more disparate in post-menopausal females with declining circulating estrogen levels. Post-menopausal women have higher rates of osteoporosis and osteoporotic-related fractures than age-matched men and estrogen deficiency has been shown to decrease the amount of newly formed bone as well as the mechanical strength of the callus [51•, 94]. Estrogen impacts the immune cell profile during fracture healing. Remarkably, in our recent study, innate immune cell populations were not different in estrogen-deficient conditions immediately following a fracture; furthermore, adaptive immune cell differences only appeared after the initial hematoma was established [51•]. By day 3 post-fracture, estrogen deficiency leads to an increase in neutrophils, T cells, B cells, and mast cells as well as an increase in their associated pro-inflammatory cytokines such as Mdk, IL-6, IL-7, and IFNγ. This low-E2-driven inflammation results in higher rates of delayed union or non-union in both post-menopausal women and aged or ovariectomized mice [92, 93, 95]. In healthy conditions, estrogen leads to an increase in VEGF expression, which inhibits osteoclast differentiation and activity, and an increase in BCL-2, which promotes osteoblast survival and differentiation [92]. Lower estrogen results in lower VEGF and therefore reduced angiogenesis and delayed bone healing [96]. Estrogen also directly regulates skeletal stem cell regeneration in both mice and humans but does not directly affect osteoclast function. Loss of function in post-menopausal women could be rescued with E2 treatments [97].

## Conclusion

It is evident that the successful healing of bone fractures is dependent upon the immune system. Signaling from immune cells is critical not only for the initial inflammation stage but also for the later soft and hard callus stages. Continued work to better understand the processes and the signals secreted by these cells will likely lead to novel therapeutic targets for improved bone fracture healing.

## Data Availability

No datasets were generated or analysed during the current study.

## Abbreviations

BM MΦ: Bone marrow–derived macrophages
CCL: Chemokine (C–C motif) ligand
CD: Cluster of differentiation
CLP: Common lymphoid progenitor
CMP: Common myeloid progenitor
CRP: C-reactive protein
CXCL: Chemokine (C-X-C motif) ligand
DC: Dendritic cell
G-CSF: Granulocyte colony-stimulating factor
GM-CSF: Granulocyte-macrophage colony-stimulating factor
IFNγ: Interferon gamma
IGF: Insulin-like growth factor
HSC: Hematopoietic stem cell
IL: Interleukin
M1: Pro-inflammatory classically activated macrophages
M2: Anti-inflammatory alternatively activated macrophages
MC: Mast cell
MCP: Monocyte chemotactic protein
M-CSF: Macrophage colony-stimulating factor
MIP: Macrophage inflammatory protein
MK: Megakaryocyte
OB: Osteoblast
OC: Osteoclast
OPG: Osteoprotegerin
RANK: Receptor activator of nuclear factor ĸ B
RANKL: Receptor activator of nuclear factor ĸ B ligand
TGFβ: Transforming growth factor-β
TNFα: Tumor necrosis factor alpha
